# Physical Activity-Related Metabolites Are Associated with Mortality: Findings from the Atherosclerosis Risk in Communities (ARIC) Study

**DOI:** 10.3390/metabo11010059

**Published:** 2021-01-19

**Authors:** Jun Xu, Guning Liu, Sheila M. Hegde, Priya Palta, Eric Boerwinkle, Kelley P. Gabriel, Bing Yu

**Affiliations:** 1 Department of Epidemiology, Human Genetics, and Environmental Sciences, School of Public Health, The University of Texas Health Science Center at Houston, Houston, TX 77030, USA; Jun.Xu.1@uth.tmc.edu (J.X.); Guning.Liu@uth.tmc.edu (G.L.); Eric.Boerwinkle@uth.tmc.edu (E.B.); 2 Division of Cardiovascular Medicine, Department of Medicine, Brigham and Women’s Hospital, Boston, MA 02115, USA; shegde@bwh.harvard.edu; 3 Division of General Medicine, Department of Medicine, Columbia University Irving Medical Center, New York, NY 10032, USA; pp2464@cumc.columbia.edu; 4 Human Genome Sequencing Center, Balor College of Medicine, Houston, TX 77030, USA; 5 Department of Epidemiology, School of Public Health, The University of Alabama at Birmingham, Birmingham, AL 35294, USA; gabrielk@uab.edu

**Keywords:** metabolomics, physical activity, mortality, metabolite risk score

## Abstract

Habitual physical activity can diminish the risk of premature death. Identifying a pattern of metabolites related to physical activity may advance our understanding of disease etiology. We quantified 245 serum metabolites in 3802 participants from the Atherosclerosis Risk in Communities (ARIC) study using chromatography–mass spectrometry. We regressed self-reported moderate-to-vigorous intensity leisure-time physical activity (LTPA) against each metabolite, adjusting for traditional risk factors. A standardized metabolite risk score (MRS) was constructed to examine its association with all-cause mortality using the Cox proportional hazard model. We identified 10 metabolites associated with LTPA (*p* < 2.04 × 10^−4^) and established that an increase of one unit of the metabolic equivalent of task-hours per week (MET·hr·wk^−1^) in LTPA was associated with a 0.012 SD increase in MRS. During a median of 27.5 years of follow-up, we observed 1928 deaths. One SD increase of MRS was associated with a 10% lower risk of death (HR = 0.90, 95% CI: 0.85–0.95). The highest vs. the lowest MRS quintile rank was associated with a 22% reduced risk of death (HR = 0.78, 95% CI: 0.62–0.94). The effects were consistent across race and sex groups. In summary, we identified a set of metabolites associated with LTPA and an MRS associated with a lower risk of death. Our study provides novel insights into the potential mechanisms underlying the health impacts of physical activity.

## 1. Introduction

Physical activity (PA) is an important modifiable lifestyle factor that can reduce the risk of premature death (i.e., death prior to the average age of death in a population) and improve the overall health status [1,2,3,4,5,6,7,8,9]. Several recent studies have demonstrated a dose–response relationship between moderate-intensity physical activity and all-cause mortality [6,9]. Physical activity-linked health benefits may be explained by various metabolic changes [10,11,12,13]. For example, it can ameliorate the excess health risk associated with adiposity [2,14,15,16,17,18,19] and it can also improve the blood lipid profile and cardiorespiratory fitness measures [20,21,22]. However, the underlying metabolic mechanisms that explain these benefits remain unclear.

Metabolomics, a type of analysis to characterize metabolic phenotypes, is a promising approach to identifying metabolic signatures associated with physical activity [23,24,25,26,27]. A study initiated in European twins has identified lipid metabolites involved in lipoprotein, cholesterol, and fatty acid pathways, which were associated with physical activity [28]. Research focusing on metabolic pathways correlated with lifestyle factors has shown that some amino acids were positively associated with physical activity [29]. For example, high levels of serine and glycine were associated with higher physical activity levels [30]. Other amino acids, such as branched-chain amino acids (BCAAs), including valine, leucine, and isoleucine, have been reported to have an inverse association with physical activity levels [31]. Some of the identified physical activity-related metabolites have been linked to disease conditions, including heart disease and type 2 diabetes [30,31,32]. A recent study focusing on community-dwelling individuals has shown extensive metabolic changes after acute exercise, which were associated with long-term mortality [33]. Those studies provide insights into disease-related pathways and have potential significance for public health practice.

Few studies have assessed the health outcomes associated with metabolites related to habitual physical activity. In addition, the available findings are primarily from European and Asian populations. In this study, we aimed to assess the association between physical activity and 245 circulating metabolites in 3802 African and European American participants of the Atherosclerosis Risk in Communities (ARIC) study and to examine the relationship between physical activity-associated metabolites and all-cause mortality during a median follow-up of 27.5 years.

## 2. Results

### 2.1. Population Characteristics

The present analysis included 3802 participants selected from the ARIC study (Figure S1); their baseline characteristics are presented in Table 1. The mean and standard deviation (SD) of ages across physical activity groups ranged from 53.52 to 53.69 and from 5.7 to 5.8, respectively. Compared to participants with poor levels of PA, those with ideal levels of PA were more likely to have lower body mass index (BMI and, systolic blood pressure (SBP), no or limited smoking habit, and lower prevalence of diabetes. During a median 27.5 years follow-up, we observed 1928 deaths.

### 2.2. Metabolites and Physical Activity

**Figure 1:**
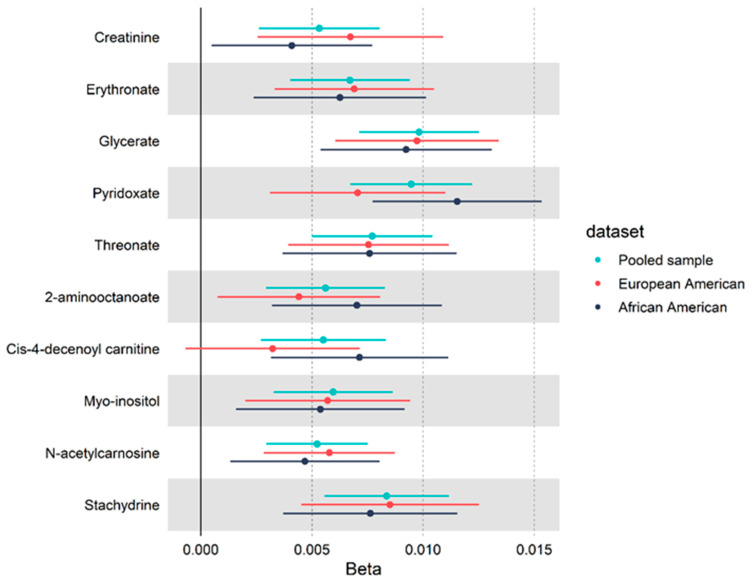
Fully adjusted association between moderate-to-vigorous-intensity leisure-time physical activity (MET·hr·wk^−1^) and 10 metabolites in 3802 participants from the Atherosclerosis Risk in Communities study.

We observed significant associations between leisure-time physical activity (LTPA) and individual metabolites. The relationship between LTPA and individual metabolites were consistent with model 1, adjusting for age, sex, race, batch, BMI, and smoking status, and with model 2, which additionally accounted for lipid, glycemic, and blood pressure variables (Table S1). In model 2 (i.e., the fully adjusted model), 10 metabolites were identified to be significantly associated with LTPA (*p* < 2.04 × 10^–4^). An increase of one metabolic equivalent of task (MET) hours per week (MET·hr·wk^−1^) in LTPA was associated with an average increase of 0.007 SD in the standardized levels of metabolites, ranging from 0.005 to 0.010 SD. (Figure 1). Those 10 metabolites were found to be involved in 6 super-pathways, including one amino acid (creatinine) pathway, three lipid (2-aminooctanoate, cis-4-decenoyl carnitine and myo-inositol) pathways, one peptide (N-acetylcarnosine) pathway, two carbohydrate (glycerate and erythronate) pathways, two cofactor/vitamin (threonate and pyridoxate) pathways, and one xenobiotic (stachydrine) pathway. Glycerate presented the largest effect size, with one MET·hr·wk^−1^ unit increase in LTPA associated with a 0.010 SD increase in glycerate levels. Modest correlations were observed across the 10 significant metabolites (median *r* = 0.37, IQR = 0.35, Figure S2). Because creatinine is a biomarker of kidney function, we conducted sensitivity analyses using the function developed by the Chronic Kidney Disease Epidemiology Collaboration to determine the estimated glomerular filtration rate (eGFR_CKDepi). The effect of LTPA was attenuated for half of the identified metabolites, but associations were not materially altered (all *p* < 0.05, Table S1). In the stratified analyses, comparable results were observed between African Americans (AAs) and European Americans (EAs), as well as among men and women (Table S2).

We then derived a metabolite risk score (MRS) by summing quintile ranks of the 10 significant metabolites and examined its relationship with the continuous and categorical LTPA measures (based on the American Heart Association (AHA) “Life’s Simple 7” physical activity metrics), separately. The raw MRS was approximately normally distributed in the entire population, as well as in race and sex groups (Figure S3). It was strongly associated with both continuous and categorical LTPA measures, as expected. A one-unit higher MET·hr·wk^−1^ in LTPA was associated with a 0.012 SD greater standardized MRS (beta = 0.012, 95% CI: 0.0010–0.015, *p* = 2.65 ×10^−20^) in the fully adjusted model. Comparing individuals in the lower category according to the AHA recommendations, participants who belonged to the intermediate and ideal categories were associated with a 0.184 SD (95% CI: 0.105−0.263, *p* = 5.34 × 10^−6^) and a 0.374 SD (95% CI: 0.302−0.446, *p* = 5.52 × 10^−24^) higher standardized MRS in the fully adjusted model. Because a moderate-to-strong correlation (r = 0.62−0.83) was observed for three metabolites (glycerate, threonate, and erythronate), we conducted a sensitivity analysis including only glycerate into the MRS, as glycerate showed the strongest variations between LTPA levels. The newly constructed MRS using 8 metabolites showed consistent association when compared to the original MRS using 10 metabolites (data not shown).

### 2.3. MRS and All-Cause Mortality

**Figure 2:**
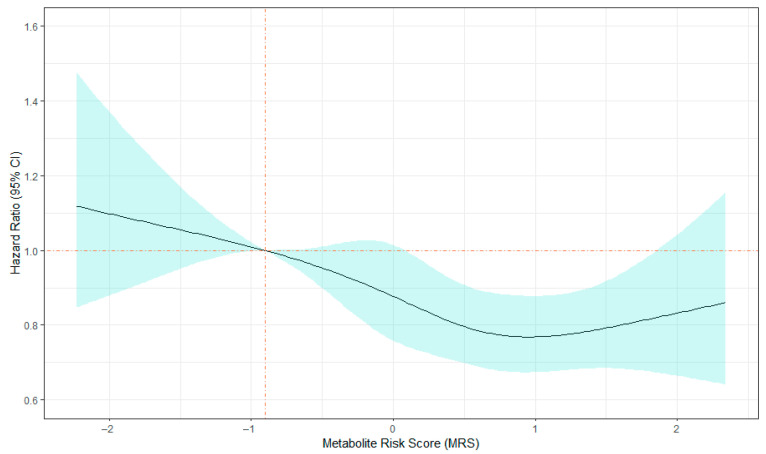
Association between standardized MRS and log hazard ratio for all-cause mortality in 3802 participants from the Atherosclerosis Risk in Communities study.

During a median of 27.5 years of follow-up, 1928 deaths were observed among the 3802 ARIC participants. The standardized MRS and its quintile ranks were significantly associated with a lower risk of all-cause mortality after adjusting for known risk factors as well as LTPA (Table 2). One SD increase in MRS was associated with a 10% lower risk of mortality (HR = 0.90, 95% CI: 0.85−0.95), and the highest MRS quintile rank was associated with a 22% lower risk of mortality when compared to the lowest quintile rank (HR = 0.78, 95% CI: 0.62–0.94). Hazard ratio trends suggested a nearly dose–response relationship with all-cause mortality (Figure 2). The mediation analysis revealed that 26.5% of the total effect of physical activity on all-cause mortality might be explained by the MRS. In the sensitivity analysis, participants without reported participation in physical activity were excluded. Participants in the resulting subsamples were on average healthier, such as including a lower proportion of smokers and having lower BMI and SBP, when compared to the entire study population (n = 1979; Table S3). The sensitivity analyses showed consistent results across race and sex groups, with high MRS associated with low risk of all-cause mortality, and the effect was independent of LTPA (Table S4).

## 3. Discussion

In a bi-racial cohort including 3802 African and European Americans, 10 circulating metabolites were identified to be significantly associated with habitual physical activity. An MRS, generated from the 10 identified metabolites, was positively associated with LTPA, and the effect was generally consistent across race and sex groups. In a prospective analysis with a median follow-up of 27.5 years, a dose–response relationship was observed, such that higher MRS levels were associated with a lower risk of all-cause mortality, and the effect persisted after additional adjustment of LTPA. Our findings illustrate the metabolic response to physical activity and its impact on mortality.

Among the 10 metabolites we identified, 4 metabolites, i.e., threonate, myo-inositol, creatinine, and cis-4-decenoyl carnitine, have been reported to be associated with physical activity. Threonate, a vitamin derivative, was positively associated with physical activity energy expenditure. [31] Threonate is a degradation product of L-ascorbate (vitamin C) involved in the ascorbate and aldarate metabolic pathway. [34] A prior study with 427 participants demonstrated that compared to nonathletes, athletes could absorb and use a larger proportion of vitamin C from diet, which may lead to higher levels of circulating ascorbic acid. Another metabolite, myo-inositol, was also positively associated with physical activity. Elevated levels of myo-inositol were found in women undergoing weight loss treatment that included behavioral targets to increase physical activity and improve diet [35]. Studies show that myo-inositol metabolism may be important for multiple cell essential functions, and abnormalities in myo-inositol metabolism were related to diabetes complications and insulin resistance [36,37,38]. Creatinine is generated mainly by muscle [39], and the level of creatinine in the blood is a result of multiple factors, including age, sex, race, diet, skeletal muscle mass. Creatinine level has been used as a measurement or indicator for muscle mass or kidney function [40]. Active individuals [41] can have higher creatinine levels compared to inactive individuals. In the present study, we found that serum creatinine was positively associated with reported LTPA. This may be explained by the fact that people who perform higher intensity physical activity are likely to have greater skeletal muscle mass. Cis-4-decenoyl carnitine is a chain-shortened product resulted from incomplete β-oxidation. Studies have shown that chain-shortened products, such as decenoyl carnitine derivatives, are generally increased by exercise training [42]. Our study has consistently shown that LTPA is associated with increased cis-4-decenoyl carnitine levels.

Six out of the 10 metabolites we found are novel for their relationships with physical activity, but the biological function of some of them remains unclear. Glycerate (also called glyceric acid), a metabolite of a colorless syrupy acid obtained from the oxidation of glycerol, is involved in glycolysis and pyruvate metabolism. In this metabolic pathway, glycerate is an intermittent reagent in the reaction to produce pyruvate. [43] When physical activity levels increase, the sequence of glycolysis reactions is vigorous [44]. As a result, the levels of glycerate may also increase [45]. We detected a positive association between glycerate and reported LTPA, and glycerate appeared as the metabolite with the largest effect. Pyridoxate is a catabolic product of vitamin B6, produced during the breakdown process of pyridoxal and excreted in urine. It is biosynthesized in a bacterial reaction related to *Escherichia coli* [46]. A recent study demonstrated that beneficial microbial species can be enhanced by physical activity, which leads to improved health in the host [47]. This suggests that the association between pyridoxate and physical activity may be a consequence of an interaction between the microbiota and the host. Pyridoxate participates in vitamin B6 metabolism. Blacks were shown to have lower vitamin B6 concentrations than non-Hispanic whites in the National Health And Nutrition Examination Survey (NHANES) [48]. A recent study has shown that higher vitamin B6 concentrations were associated with a lower risk of mortality [49]. The compound 2-aminooctanoate is an organic molecule that belongs to the amino acid class. It can be detected in cow’s milk, which makes it one of the indicators for milk consumption [50]. Previous dietary studies have shown that decreased levels of 2-aminooctanoate are related to the consumption of navy bean and/or rice bran [51]. However, the possible mechanism linking 2-aminooctanocte metabolism and physical activity is not clear.

Few studies have examined the relationship between physical activity-associated metabolites on all-cause mortality. Here, we demonstrated that the MRS was positively associated with physical activity and with a protective effect on all-cause mortality. Two metabolites involved in the MRS have been reported to increase the risk of all-cause mortality: erythronate was associated with higher overall mortality in male smokers, and creatinine was related to increased overall mortality in males with mild renal deficiency [52,53]. This inversed effect compared to our findings is possibly due to the specific populations studied. One metabolite that we found influences the MRS, N-acetylcarnosine, has been shown to have a protective effect on all-cause mortality in participants with chronic kidney disease [54]. Previous studies show that physical activity has a graded effect on all-cause mortality [1,3]. We found a similar trend for LTPA-derived MRS, with a higher MRS associated with a lower risk of mortality, which may provide insights into the amount of LTPA needed to get benefit. Previous studies have found PA to be inversely associated with triglycerides concentrations [55] and positively associated with high-density lipoproteins (HDL) concentrations [56]. In our study, we did not observe such effects in the race-pooled analysis. When we stratified the analysis by race groups, the relationships between PA, triglycerides, and HDL–cholesterol (C) were consistent with previous studies in European Americans.

Our study has several strengths. We conducted the analyses in a large and well-characterized cohort of African and European Americans. This allowed us to investigate the relationship between physical activity and metabolites in AAs, a population with higher risk of premature death, and compare/contrast the findings to those for EAs. We utilized untargeted metabolomic profiling to capture a wide range of circulating metabolites, which provided us with a comprehensive view of metabolism. Our cohort has been followed up for more than 27 years, which offered us the opportunity to explore the long-term health impact of physical activity-related metabolites ascertained at midlife. Our study also has a few limitations. Physical activity information was self-reported rather than objectively measured, which may be prone to recall bias and social desirability. However, previous studies have agreed on the high reliability and validity of the Baecke questionnaire we implemented [57,58,59,60]. The metabolite quantification protocol we used produced semi-quantitative levels. Future studies are warranted to quantify those metabolites using standard assays. In addition, we studied a variety of circulating metabolites, but other small molecules (i.e., lipoproteins) were not captured. Such small molecules may provide additional insights into the mechanisms linking physical activity and mortality. Our metabolomic profiling was conducted cross-sectionally, and our findings were focused on a middle-aged population. Further longitudinal analysis is warranted to assess the metabolic alterations associated with physical activity and other lifestyle changes, and their impact on mortality. Our serum samples were stored for more than 20 years, and prolonged storage of blood samples may lead to altered metabolite levels. However, we consider that the alteration of metabolite levels was systematic among all participants, and a similar design has been used in other metabolomics studies [52]. Finally, although we observed similar effects in African and European Americans, generalizing our findings to other populations requires further investigation.

## 4. Materials and Methods

### 4.1. Study Participants

The ARIC study is an ongoing prospective population-based cohort with 15,792 enrolled participants between 45 and 64 years of age from four communities in United States (Forsyth County, North Carolina; Jackson, Mississippi; suburbs of Minneapolis, Minnesota; and Washington County, Maryland). Seven visits were conducted (visit 1: 1987–1989, visit 2: 1990–1992, visit 3: 1992–1995, visit 4: 1996–1998, visit 5: 2011–2013, visit 6: 2016–2017, and visit 7: 2018–2019). A comprehensive description of the study design and methods was published previously [61]. In the present analysis, we studied 3802 participants with available physical activity and metabolomic measures at visit 1 (1987–1989). Written informed consent was obtained from all ARIC study participants, and the ARIC study was approved by the institutional review board at the participating institutions.

### 4.2. Assessment of Physical Activity, All-Cause Mortality, and Covariates

In the ARIC study, LTPA was assessed using a modified version of the Baecke physical activity questionnaire, which exploits a past-year recall time frame [62,63,64]. The sports and leisure section of the modified Baecke questionnaire prompts participants to report up to four most common activity types done over the past 12 months. For each activity type reported, additional information on the duration (hours per week) and frequency (number of weeks within one month) is ascertained. The reliability and validity of the Baecke questionnaire was published elsewhere [59,60]. While the traditional Baecke questionnaire scoring of summary estimates results in an index score ranging from 1 (low) to 5 (high), the sports and leisure domain was transformed and calculated as metabolic equivalent of task (MET) hours per week (MET·hr·wk^−1^), given it is a more physiologically meaningful estimate that can be extrapolated to a variety of public health metrics. Each activity type was assigned a MET value ranging from 1 to 12 METs, based on the 2011 Compendium of Physical Activities [65]. For each activity type reported, MET·hr·wk^−1^ was estimated over the past year by multiplying the intensity, frequency, and duration, aggregated across all activity (up to four) types reported to quantify leisure-time physical activity. Moderate-to-vigorous LTPA (all reported activity types ≥ 3 METs) was also expressed categorically, based on recommendations set forth by the 2018 Physical Activity Guidelines for Americans and the American Heart Association’s (AHA) “Life’s Simple 7” physical activity metrics. Categories include poor (0 min·wk^−1^), intermediate (1 to 149 min·wk^−1^ of moderate-intensity, 1 to 74 min·wk^−1^ of vigorous-intensity, or 1 to 149 min·wk^−1^ of moderate-to-vigorous-intensity activity), or ideal (≥150 min·wk^−1^ of moderate-intensity, ≥75 min·wk^−1^ vigorous-intensity, or ≥150 min·wk^−1^ of moderate- plus vigorous-intensity activity).

In the ARIC study, we followed the vital status of each participant from visit 1 until 31 December 2017. For those who died during the follow-up, death information was obtained from multiple sources, including record of annual cohort follow-up (and semi-annual follow-up since 2012), community-wide hospital surveillance, records from national and local death registries, and death certificates. Trained medical abstractors reviewed the death certificates to determine the causes of death. In the present study, our primary outcome was all-cause mortality.

Covariates of interest, including age, sex, race, and others, were obtained at the visit 1 interview and physical examination. BMI (kg/m^2^) was computed using measured weight (kg) and standing height (m). Cigarette smoking status was categorized as current, former, or never smoker. Seated SBP was examined three times by a random-zero mercury sphygmomanometer, and the average number of the last two measurements was included in the analysis. Antihypertensive medication usage information was collected from both self-report questionnaires and medication inventory. The criteria of prevalent diabetes included fasting blood glucose ≥126 mg/dL, non-fasting glucose ≥200 mg/dL, and a self-reported usage of antidiabetic medications or insulin or a self-reported diagnosis of diabetes by a physician. The concentrations of serum HDL–cholesterol, cholesterol, and triglycerides were assessed by standardized enzymatic approaches. The estimated glomerular filtration rate was calculated using the equation developed by the Chronic Kidney Disease Epidemiology Collaboration (eGFR_CKDepi) [66]. Prevalent cardiovascular disease was demarcated as self-reported prevalent cases of coronary heart disease, stroke, or heart failure.

### 4.3. Metabolomic Profiling

Metabolomic profiling was performed in 1880 African Americans in 2010 and 2152 African and European Americans in 2014 using serum samples collected at visit 1, which had been stored at −70 °C since their initial collection. All samples were assayed using untargeted liquid or gas chromatography–mass spectrometry by Metabolon, Inc. (Durham, NC, USA) [67,68]. The Pearson correlation coefficient was calculated in a subset of 97 samples assayed in both 2010 and 2014 [69]. For the present study, metabolites were excluded if (1) they were only detected in one batch; (2) the proportion of the missing values was higher than 25% in either batch; (3) *r* was less than 0.3. There were 384 named metabolites assayed in both batches. Two hundred and forty-five named metabolites were included in the analysis after applying the exclusion criteria. These metabolites are involved in eight super-pathways: amino acid, carbohydrate, cofactors and vitamins, energy, lipid, nucleotide, peptide, and xenobiotics. Metabolites with detected values outside the 1st to the 99th percentile in each batch were winsorized to the 1st or the 99th percentile. Individuals with missing information on metabolite values were imputed to the lowest value of that metabolite in each batch. The natural log-transformed metabolites were normalized by their means and SDs prior to the analysis.

### 4.4. Statistical Analysis

For this study, we excluded participants with missing covariates of interest (*n* = 230), leaving 3802 participants for the analyses (Figure S1). We applied linear regression models to examine the associations of LTPA (MET) with 245 metabolites using two consecutive models: model 1 with adjustments for age, sex, race, smoking status, BMI, and batch; and model 2 with additional adjustments for HDL, triglycerides, total cholesterol, glucose, diabetes status, SBP, and antihypertensive medication use [16,69,70]. The significance level was defined as *p* < 2.04 × 10^−4^ using Bonferroni correction to account for 245 metabolites. We conducted a sensitivity analysis by further adjusting for eGFR_CKDepi for the identified significant metabolites. We performed race and sex stratified analyses using the same models to investigate potential effect modifications. To examine the correlation between selected metabolites, we calculated the Spearman correlation coefficient (*r*).

For metabolites identified as associated with LTPA, we generated an MRS to represent their overall effect. For each metabolite, we ranked participants into quintiles; thus, each participant had a ranked score ranging from one to five. For each participant, the MRS was obtained by summing the ranked scores of each metabolite. The MRS was then standardized by its mean and SD and was further divided into quintiles for association analyses [71].

We conducted two analyses based on the derived scores. We first regressed AHA categories of LTPA against the MRS and its quintile ranks using linear regressions adjusting for the same covariates as model 2. We next examined the prospective associations of MRS and its quintile ranks with incident all-cause mortality using the Cox Proportional Hazard model, adjusting for age, sex, race, batch, BMI, SBP, diabetes status, smoking status, HDL, triglycerides, total cholesterol, antihypertensive medication use, prevalent cardiovascular disease, eGFR_CKDepi [66] and LTPA levels. The proportional hazards assumption was assessed by a goodness-of-fit test based on weighted residuals, and no violation was observed. We performed a sensitivity analysis by excluding those participants who did not report participation in any physical activity (i.e., zero MET·hr·wk^−1^) using the same model. We further conducted a model-based mediation analysis [72] to estimate the proportion of the effect of physical activity on all-cause mortality that was mediated through MRS.

All statistical analyses were conducted by using R 3.5.2 (R Development Core Team, R Foundation for Statistical Computing, Vienna, Austria (http://www.r-project.org)).

## 5. Conclusions

In conclusion, we identified 10 metabolites that were associated with physical activity and demonstrated that an MRS constructed from the 10 metabolites was associated with the risk of all-cause mortality. Our results suggest potential pathways explaining the health benefits of physical activity and provide additional insights into the relationship between physical activity and all-cause mortality.

## Figures and Tables

**Table 1:** Baseline participant characteristics in the Atherosclerosis Risk in Communities (ARIC) study, 1987–1989 (*n* = 3802), stratified by physical activity (PA) categories.

Characteristics	Poor PA *n* = 1823	Intermediate PA *n* = 805	Ideal PA *n* = 1174	*p* Value *
Age, years	53.52 (5.7)	53.63 (5.7)	53.69 (5.8)	0.41
African Americans, *n* (%)	1375 (75.4)	434 (53.9)	535 (45.6)	<0.001
Male, *n* (%)	649 (35.6)	281 (34.9)	594 (50.6)	<0.001
BMI, kg/m^2^	29.64 (6.4)	28.33 (5.4)	27.63 (5.0)	<0.001
Smoking				
Never smoker, *n* (%)	835 (45.8)	397 (49.3)	453 (38.6)	<0.001
Former smoker, *n* (%)	427 (23.4)	219 (27.2)	418 (35.6)	
Current smoker, *n* (%)	561 (30.8)	189 (23.5)	303 (25.8)	
LTPA, MET⋅hr⋅wk^−1^	0 (0)	5.37 (3.0)	22.12 (11.3)	<0.001
Diabetes, *n* (%)	291 (16.0)	88 (10.9)	138 (11.7)	<0.001
Cardiovascular disease, *n* (%)	190 (10.4)	80 (9.9)	146 (12.4)	0.13
eGFR, mL/min/1.73 m^2^	101.56 (18.4)	98.70 (17.1)	95.67 (17.0)	<0.001
HDL, mmol/L	1.3 (0.4)	1.38 (0.4)	1.37 (0.4)	0.15
Triglycerides, mmol/L	1.26 (0.6)	1.34 (0.7)	1.34 (0.7)	<0.001
Total cholesterol, mmol/L	5.53 (1.1)	5.58 (1.1)	5.53 (1.1)	0.92
SBP, mmHg	127.06 (21.8)	123.60 (20.6)	121.70 (19.5)	<0.001
Death, *n* (%)	980 (53.8)	376 (46.7)	572 (48.7)	0.001
MRS	28.65 (7.3)	30.32 (7.2)	31.84 (7.4)	<0.001

BMI, body mass index; LTPA, moderate-to-vigorous-intensity physical activity; MET, metabolic equivalent; eGFR, estimated glomerular filtration rate; HDL, high-density lipoprotein; SBP, systolic blood pressure; MRS, metabolite risk score. For continuous variables, mean values and standard deviation are shown. For categorical variables, numbers are given as frequency and percentage. * ANOVA and Chi-square were performed to obtain statistical significance for continuous and categorical variables, respectively.

**Table 2:** Associations between the standardized metabolite risk score (MRS), moderate-to-vigorous-intensity leisure-time physical activity (LTPA) and incident all-cause mortality.

Physical Activity *	β (95% CI)	*p* Value
MRS and LTPA	0.012 (0.0010, 0.015)	2.65 × 10^−20^
MRS and 2018 Physical Activity Guidelines		
Intermediate vs. poor	0.184 (0.105, 0.263)	5.34 × 10^−6^
Ideal vs. poor	0.374 (0.302, 0.446)	5.52 × 10^−24^
All-cause mortality ^†^	Hazard Ratio (95% CI)	*p* Value
MRS (per SD change)	0.90 (0.85, 0.95)	3.86 × 10^−5^
MRS quintiles		
Q2 vs. Q1	0.95 (0.83, 1.09)	0.48
Q3 vs. Q1	0.85 (0.74, 0.98)	0.03
Q4 vs. Q1	0.76 (0.66, 0.88)	0.0002
Q5 vs. Q1	0.78 (0.67, 0.92)	0.002

* Adjustment included age, sex, race, batch, smoking status, body mass index, glucose, diabetes, systolic blood pressure, high-density lipoprotein cholesterol, total cholesterol, triglycerides, hypertension, and antihypertensive medications. ^†^ Adjustment included age, sex, race, batch, smoking status, body mass index, diabetes, systolic blood pressure, high-density lipoprotein cholesterol, total cholesterol, triglycerides, antihypertensive medications, moderate-to-vigorous-intensity leisure-time physical activity, estimated glomerular filtration rate, and cardiovascular diseases status.

## Data Availability

The full results presented in this study are available on request from the corresponding author, and the data analyzed in this study are available on request from the ARIC study https://sites.cscc.unc.edu/aric/distribution-agreements.

## Supplementary Materials

The following are available online at https://www.mdpi.com/2218-1989/11/1/59/s1, Figure S1: Flow chart of participant selection, Figure S2: Pair-wise correlation for the 10 metabolites associated with moderate-to-vigorous-intensity leisure-time physical activity (LTPA). The size of the circle is proportional to the correlation coefficients between two metabolites, Figure S3: Distribution of raw metabolite risk score by sex group (left) and race group (right); Table S1: Association between metabolites and moderate-to-vigorous-intensity leisure-time physical activity (LTPA) in ARIC study participants, Table S2: Association between metabolites and moderate-to-vigorous-intensity leisure-time physical activity (LTPA) stratified by a) race and b) sex in ARIC study participants, Table S3: Characteristics for participants with reported non-zero physical activity, Table S4: Associations between standardized metabolite risk score and incident all-cause mortality in the sensitivity analysis.
