# Supplementary material for: Physical Activity-Related Metabolites Are Associated with Mortality: Findings from the Atherosclerosis Risk in Communities (ARIC) Study
Source: Metabolites. 2021 Jan 19;11(1):59. doi: 10.3390/metabo11010059 (PMC7835806; doi:10.3390/metabo11010059)

Supplemental Materials

Supplemental Table S1. Association between metabolites and moderate-to-vigorous-intensity leisure-time physical activity (LTPA) among ARIC study participants.

metabolites	Model 1			Model 2			Model 3		
	beta	se	p	beta	se	p	beta	se	p
Creatinine	0.005	0.001	0.0002	0.005	0.001	0.0001	0.003	0.001	0.01
Erythronate	0.007	0.001	1.54×10^{-06}	0.007	0.001	1.01×10^{-06}	0.006	0.001	2.96×10^{-05}
Glycerate	0.010	0.001	3.43×10^{-13}	0.010	0.001	1.06×10^{-12}	0.010	0.001	1.88×10^{-13}
Pyridoxate	0.010	0.001	1.09×10^{-11}	0.009	0.001	1.44×10^{-11}	0.009	0.001	6.21×10^{-10}
Threonate	0.008	0.001	1.19×10^{-08}	0.008	0.001	2.51×10^{-08}	0.008	0.001	4.27×10^{-08}
2-Aminooctanoate	0.005	0.001	0.0001	0.006	0.001	3.95×10^{-05}	0.005	0.001	0.0001
Cis-4-decenoyl carnitine	0.005	0.001	0.0003	0.005	0.001	0.0001	0.004	0.001	0.002
Myo-Inositol	0.006	0.001	6.06×10^{-05}	0.006	0.001	1.31×10^{-05}	0.004	0.001	0.002
N-Acetylcarnosine	0.005	0.001	0.0001	0.005	0.001	7.25×10^{-06}	0.003	0.001	0.001
Stachydrine	0.008	0.001	4.60×10^{-08}	0.008	0.001	5.33×10^{-09}	0.008	0.001	3.27×10^{-08}

Model 1: linear regression with adjustments for age, sex, race, smoking status, body mass index, and batch.
Model 2: linear regression with additional adjustment for glucose, diabetes, systolic blood pressure, high-density lipoprotein cholesterol, total cholesterol, triglycerides, systolic blood pressure, and antihypertensive medications.
Model 3: linear regression with additional adjustment for eGFR.

Supplemental Table S2. Association between metabolites and moderate-to-vigorous-intensity leisure-time physical activity (LTPA) stratified by a) race and b) sex among ARIC study participants.

a)			Pooled sample			African Americans			European Americans		
Biochemical	Super Pathway	HMDB	beta	se	p	beta	se	p	beta	se	p
Creatinine	Amino Acid	HMDB00562	0.005	0.001	0.0001	0.004	0.002	0.026	0.007	0.002	0.002
N-Acetylcarnosine	Peptide	HMDB12881	0.005	0.001	7.25×10^{-06}	0.005	0.002	0.006	0.006	0.001	0.0001
Glycerate	Carbohydrate	HMDB00139	0.010	0.001	1.06×10^{-12}	0.009	0.002	2.77×10^{-06}	0.010	0.002	2.52×10^{-07}
Erythronate	Carbohydrate	HMDB00613	0.007	0.001	1.01×10^{-06}	0.006	0.002	0.007	0.007	0.002	0.0002
2-Aminooctanoate	Lipid	HMDB00991	0.006	0.001	3.95×10^{-05}	0.007	0.002	0.0003	0.004	0.002	0.018
Cis-4-Decenoyl Carnitine	Lipid	#N/A	0.005	0.001	0.0001	0.007	0.002	0.0005	0.003	0.002	0.106
Myo-Inositol	Lipid	HMDB00211	0.006	0.001	1.31×10^{-05}	0.005	0.002	0.005	0.006	0.002	0.003
Threonate	Cofactors and Vitamins	HMDB00943	0.008	0.001	2.51×10^{-08}	0.008	0.002	0.0001	0.007	0.002	4.74×10^{-05}
Pyridoxate	Cofactors and Vitamins	HMDB00017	0.009	0.001	1.44×10^{-11}	0.011	0.002	3.37×10^{-09}	0.007	0.002	0.0005
Stachydrine	Xenobiotics	HMDB04827	0.008	0.001	5.33×10^{-09}	0.008	0.002	0.0001	0.008	0.002	3.23×10^{-05}
Adjusting for age, gender, race, center-batch, smoking status, body mass index, glucose, diabetes, systolic blood pressure, high-density lipoprotein cholesterol, total cholesterol, triglycerides, systolic blood pressure, and antihypertensive medications											

b)			Males			Females		
Biochemical	Super Pathway	HMDB	beta	se	p	beta	se	p
Creatinine	Amino Acid	HMDB00562	0.006	0.002	0.0003	0.004	0.002	0.078
N-Acetylcarnosine	Peptide	HMDB12881	0.007	0.001	2.74×10^{-06}	0.002	0.002	0.169
Glycerate	Carbohydrate	HMDB00139	0.009	0.002	5.19×10^{-07}	0.010	0.002	3.65×10^{-07}
Erythronate	Carbohydrate	HMDB00613	0.007	0.002	0.0002	0.006	0.002	0.002
2-Aminooctanoate	Lipid	HMDB00991	0.007	0.002	6.5×10^{-05}	0.004	0.002	0.078
Cis-4-Decenoyl Carnitine	Lipid	#N/A	0.005	0.002	0.014	0.006	0.002	0.006
Myo-Inositol	Lipid	HMDB00211	0.005	0.002	0.003	0.006	0.002	0.002
Threonate	Cofactors and Vitamins	HMDB00943	0.007	0.002	0.0005	0.009	0.002	9.53×10^{-06}
Pyridoxate	Cofactors and Vitamins	HMDB00017	0.008	0.002	7.82×10^{-06}	0.012	0.002	9.91×10^{-08}
Stachydrine	Xenobiotics	HMDB04827	0.008	0.002	1.5×10^{-05}	0.008	0.002	9.73×10^{-05}
Adjusting for age, gender, race, batch, smoking status, body mass index, glucose, diabetes, systolic blood pressure, high-density lipoprotein cholesterol, total cholesterol, triglycerides, systolic blood pressure, and antihypertensive medications								

Supplemental Table S3 Characteristics for participants with reported non-zero physical activity.

Characteristics	African Americans N = 969	European American N = 1010	*p* value
Age, years	52.7 (5.7)	54.6 (5.8)	1.76×10^{-13}
Male, n (%)	397 (41.0)	478 (48.2)	0.005
BMI, kg/m^2	29.0 (5.5)	26.9 (4.6)	2.97×10^{-20}
Smoking			
Never smoker, n (%)	455 (47.0)	395 (39.1)	5.02×10^{-04}
Former smoker, n (%)	253 (26.1)	384 (38.0)	1.90×10^{-08}
Current smoker, n (%)	261 (26.9)	231 (22.9)	0.04
LTPA, MET·hr·wk^{-1}	14.32 (11.94)	16.25 (12.23)	3.73×10^{-04}
Meeting 2018 Physical Activity Guidelines			3.17×10^{-04}
Ideal physical activity, n (%)	535 (55.2)	639 (63.3)	
Intermediate physical activity, n (%)	434 (44.8)	371 (36.7)	
Diabetes, n(%)	159(16.4)	67(6.63)	1.34×10^{-11}
MRS	29.1 (7.5)	30.8 (7.2)	2.85×10^{-07}
Cardiovascular disease, n(%)	108 (11.1)	118 (11.7)	0.76
eGFR, mL/min/1.73m^2	102.9 (17.8)	91.2 (14.2)	9.62×10^{-55}
HDL, mg/dL	1.4 (0.4)	1.3 (0.4)	7.24×10^{-10}
Triglycerides, mg/dL	1.2 (0.6)	1.5 (0.7)	2.86×10^{-21}
Total cholesterol, mg/dL	5.5 (1.2)	5.6 (1.0)	0.34
Systolic Blood Pressure, mmHg	128.3(21.7)	118.8(18.3)	9.97×10^{-16}
Death, n (%)	468 (48.3)	480 (47.5)	0.76

Note*:* BMI Body mass index, MET Metabolic Equivalent, MRS metabolite risk score, HDL high-density lipoprotein, eGFR estimated glomerular filtration rate.
For continuous variables, mean values and standard deviation are shown. For categorical variables, numbers are given as frequency and percentage.

Supplemental Table S4. Associations between standardized MRS and incident all-cause mortality in the sensitivity analysis.

	Pooled sample		Male		Female		African Americans		European Americans	
	HR (95% CI)	P Value	HR (95% CI)	P	HR (95% CI)	P	HR (95% CI)	P	HR (95% CI)	P
MRS (per SD)	0.817 (0.759, 0.879)	6.4 x 10^{-8}	0.836 (0.752, 0.929)	0.0009	0.789 (0.710, 0.878)	1.24 x 10^{-5}	0.841 (0.761, 0.929)	0.000 6	0.780 (0.698, 0.871)	1.02 x10^{-05}
MRS quartiles										
Q2 vs Q1	0.787 (0.646, 0.961)	0.018	0.871 (0.643, 1.179)	0.371	0.747 (0.570, 0.979)	0.035	0.807 (0.617, 1.056)	0.119	0.774 (0.570, 1.051)	0.101
Q3 vs Q1	0.710 (0.581, 0.867)	0.0008	0.746 (0.548, 1.015)	0.062	0.679 (0.518, 0.890)	0.005	0.722 (0.547, 0.953)	0.022	0.661 (0.491, 0.890)	0.006
Q4 vs Q1	0.584 (0.474, 0.720)	4.75 x 10^{-7}	0.649 (0.473, 0.890)	0.007	0.527 (0.393, 0.706)	1.79 x 10^{-5}	0.609 (0.448, 0.827)	0.001	0.544 (0.401, 0.738)	9.2 x10^{-05}
Q5 vs Q1	0.600 (0.481, 0.748)	5.93 x 10^{-6}	0.629 (0.460, 0.861)	0.004	0.567 (0.405, 0.795)	0.0.001	0.643 (0.474, 0.872)	0.004	0.534 (0.384, 0.742)	0.0002
Adjusting for age, sex, race, batch, smoking status, body mass index, diabetes, systolic blood pressure, high-density lipoprotein cholesterol, total cholesterol, triglycerides, antihypertensive medications, moderate-to-vigorous-intensity physical activity, estimated glomerular filtration rate, and cardiovascular disease status.										

Supplemental Figure S1. Flow chart of participant selection.

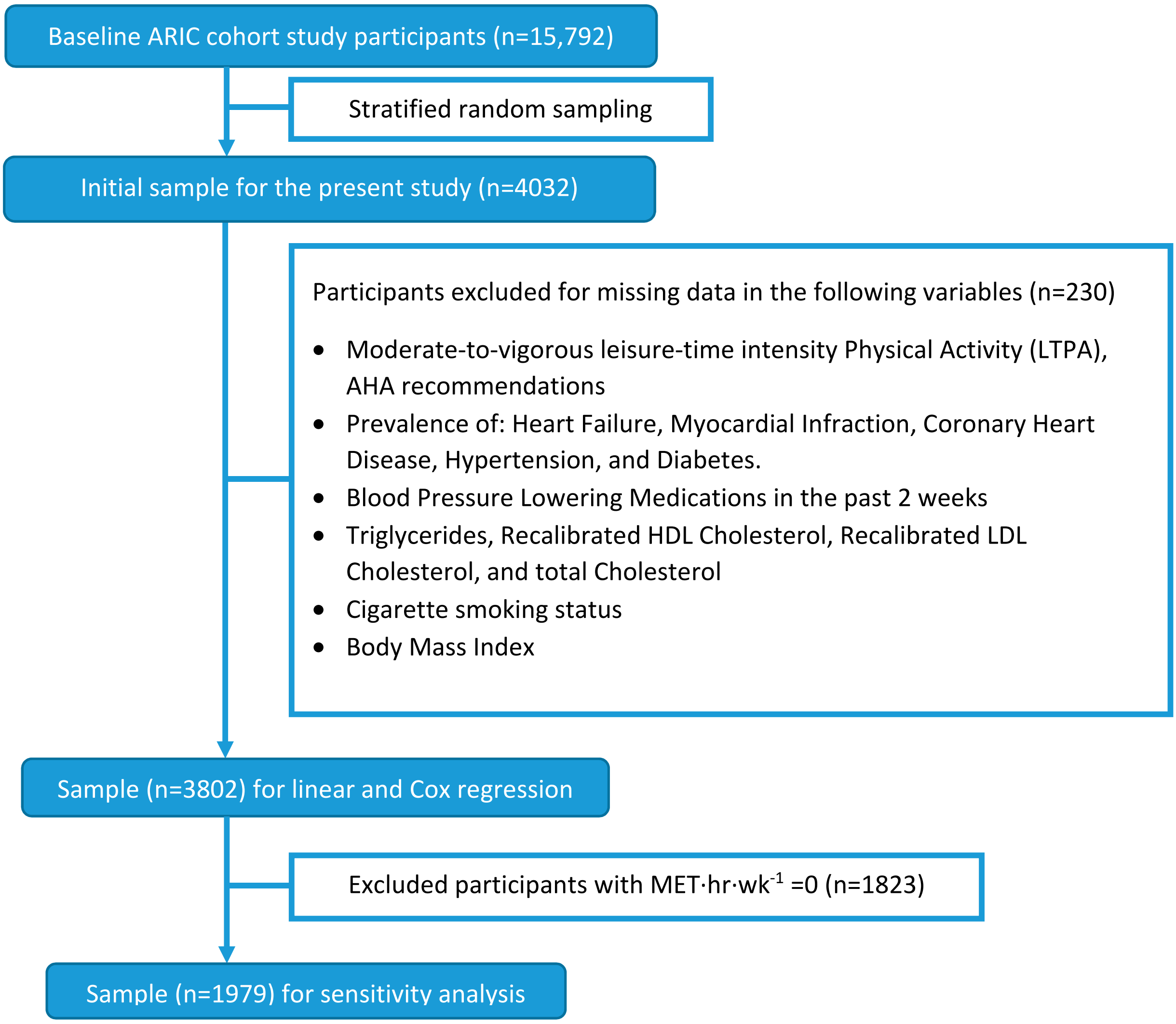

Supplemental Figure S2. Pair-wise correlation for the 10 metabolites associated with moderate-to-vigorous-intensity leisure-time physical activity (LTPA). The size of the circle is proportional to the correlation coefficients between two metabolites.

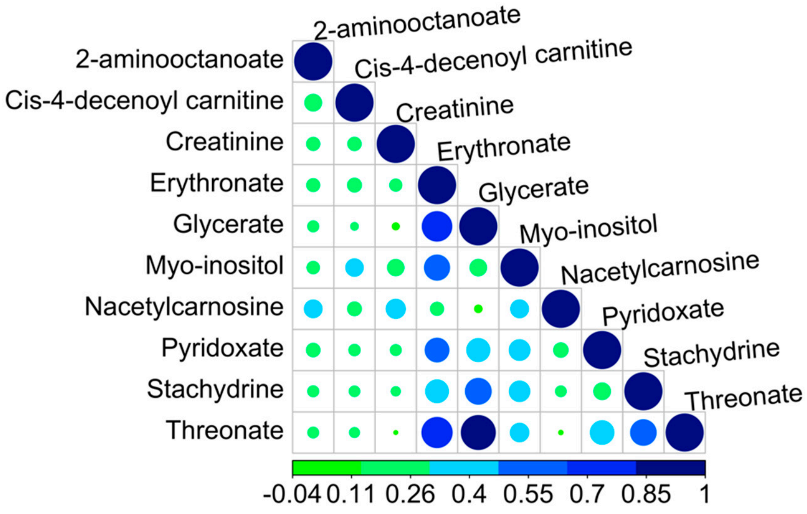

Supplemental Figure S3. Distribution of raw metabolite risk score by sex group (left) and race group (right).

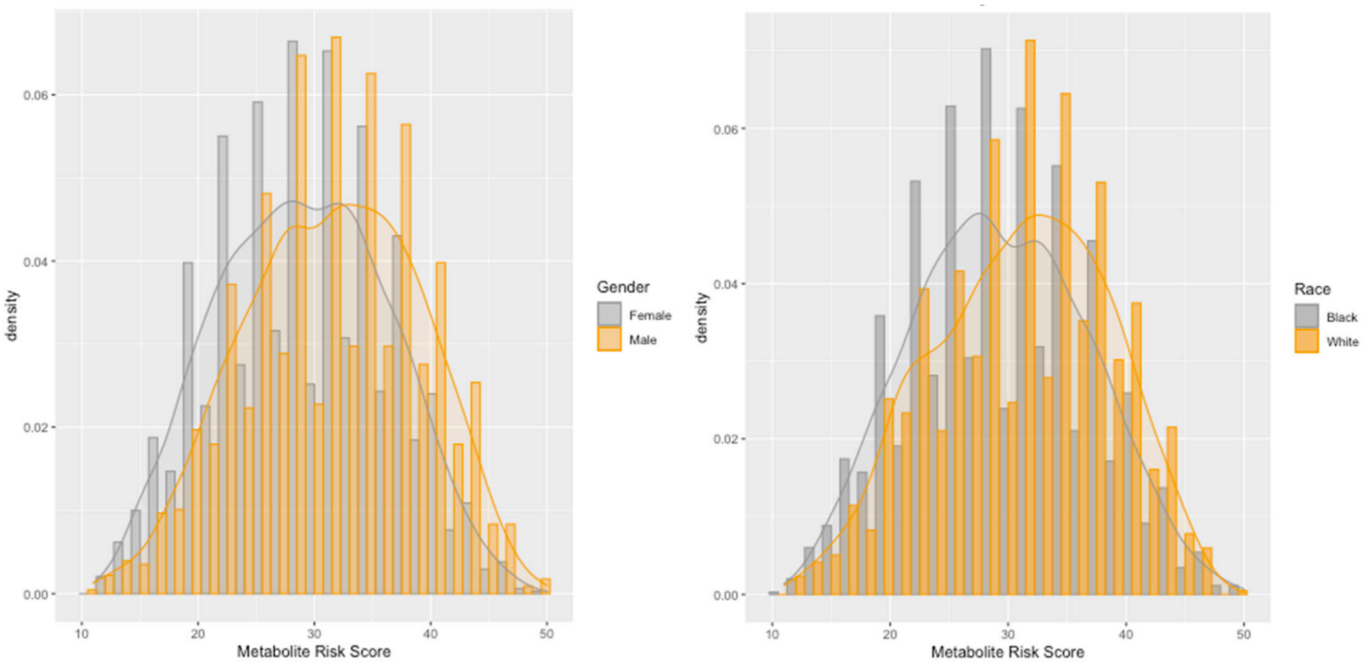

Supplement: Supplementary file 1 [file metabolites-11-00059-s001.pdf]
